# Crystal structure of the cage derivative penta­cyclo­[5.4.0.0^2,6^.0^3,10^.0^5,9^]undeca-8,11-dione ethyl­ene di­thio­ketal

**DOI:** 10.1107/S1600536814020790

**Published:** 2014-09-24

**Authors:** Sambasivarao Kotha, Nampalli Sreenivasachary, Deepak Deodhar, Mobin Shaikh

**Affiliations:** a Department of Chemistry, Indian Institute of Technology - Bombay, Powai, Mumbai 400 076, India

**Keywords:** crystal structure, bis-ketal, penta­cyclo­undecane cage derivative

## Abstract

The title penta­cyclo­undecane cage derivative, C_13_H_14_OS_2_, was identified during a thio­ketalization reaction. The reaction selectively gave the title compound and the product corresponding to bis-ketal was not formed. The title compound exhibits unusual C*sp* ^3^—C*sp* ^3^ single bond lengths ranging from 1.495 (3) to 1.581 (2) and strained bond angles as small as 89.29 (12) and as large as 115.11 (11)°.

## Abstract 2

The penta­cyclo­undecane cage derivative exhibits unusual C*sp* ^3^—C*sp* ^3^ single bond lengths ranging from 1.495 (3) to 1.581 (2) and strained bond angles as small as 89.29 (12) and as large as 115.11 (11)°.

## Chemical context   

Caged mol­ecules have found utility in various fields of science such as medicine, high energy materials and complex natural product synthesis. The high symmetry, rigid geometry and inherent strain present in these mol­ecules make them theoretically inter­esting and synthetically challenging mol­ecular frames (Marchand, 1989 ▶; Mehta *et al.*, 1997 ▶).
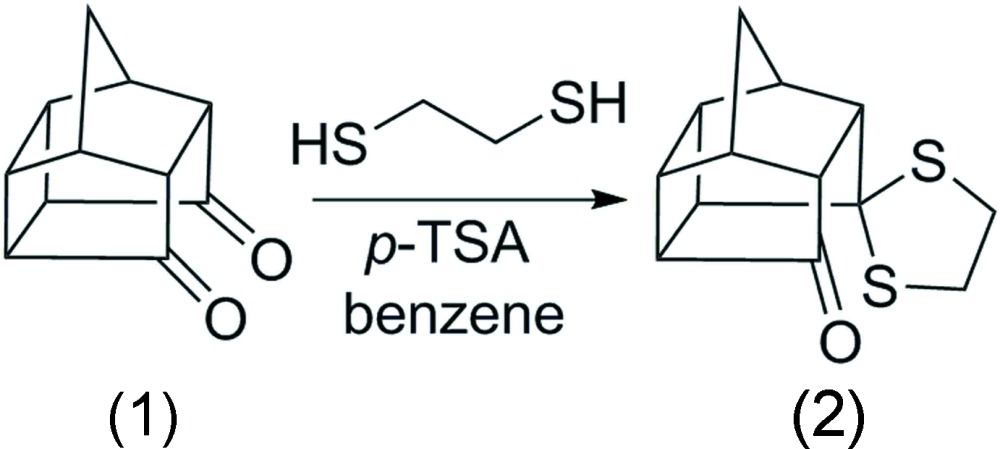


**Figure 1:**
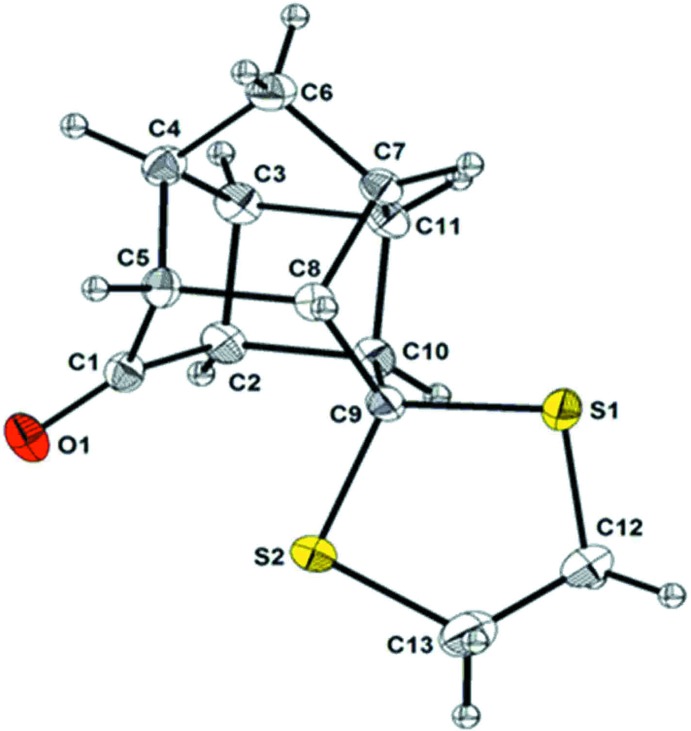
*ORTEP* diagrams of (2) showing the atom-numbering scheme. Displacement ellipsoids are drawn at the 50% probability level.

In contribution to the ongoing research in the versatile area of pentacyclo[5.4.0.0^2,6^.0^3,10^.0^5,9^]undecane-8,11-dione (PCUD) cage compounds, we report the crystal structure of an unsymmetrically substituted di­thio­ketal derivative (2). The dione (1) was treated with 1,2-ethane­dithiol using benzene as a solvent under reflux temperature. The reaction selectively gave (2), a mono-substituted product rather than the symmetrically di-substituted product or rearranged product (Fig. 1 ▶). The title compound (2) is known (Saures *et al.*, 1983 ▶; Mlinarić-Majerski *et al.*, 1998 ▶) but its crystal structure has not been reported to date.

## Structural commentary   

The cage skeleton of (2) can be described as a fusion of four five-membered rings and one four-membered and one six-membered ring. The title compound is unsymmetrically substituted at the mouth of the cage, with a ketone substituent at atom C1 and a di­thio­ketal substituent at atom C9 classifying the mol­ecule as a spiro-compound (Fig. 1 ▶). The five-membered rings C3/C4/C6/C7/C11 and C4–C7/C8/C5 each adopt an almost ideal envelope conformation (flap atom C6). The di­thio­ketal ring, S1/C9/S2/C13/C12, also adopts an envelope conformation (flap atom C9). The remaining two rings, C2–C4/C5/C1 and C7–C8 are twisted on C1–C5 and C7–C11, respectively. The tetra­hedral bond angle C3—C2—C10 is the most strained, corresponding to the smallest angle [89.29 (12)°]. The C8—C9—S2 angle [115.11 (11)°] is found to be the largest one. The deviations from the standard value of 109.5° are considerable.

Previous studies showed that PCU-caged compounds normally display C—C bond lengths which deviate from expected value of 1.54 Å (Bott *et al.*, 1998 ▶: Linden *et al.*, 2005 ▶; Kruger *et al.*, 2005 ▶; Flippen-Anderson *et al.* 1991 ▶). The structure of (1) also exhibits unusual C*sp* ^3^—C*sp* ^3^ single bond lengths ranging from 1.507 (2) to 1.581 (2) Å. In compound 2, the bond C5—C8, which is parallel and immediately adjacent to the C1–C9 axis was found to be longest at 1.581 (2) Å. Similarly, the bonds C2—C3, C3—C4, C4—C5 and C10—C11 were also found to exceed the expected value of 1.54 Å. The bonds C4—C6, C6—C7, C1—C5 and C1—C2 are short and deviate from the standard value. In the four-membered ring, one side is significantly longer [C2—C10, 1.57 (2) Å] than the remaining sides which are statistically equivalent. The C2—C10 and C5—C8 bonds [1.58 Å (2)] are the longest in (2) and similar to the same bonds in (1) [1.585 (4)–1.592 (4) Å; Linden *et al.,* 2005 ▶].

The presence of C—S bonds in (2) reveals the loss of coupling of one *sp* ^2^ carbon atom in the parent diketone (1). The distance between the carbons C10 and C9 bearing di­thio­ketal ring is found to be considerably longer [1.533 (2) Å] than the carbons C1 and C2 [1.507 (2) Å] bearing the carbonyl group.

## Synthesis and crystallization   

Preparation of compound (2): To a stirred suspension of dione (1) (630 mg, 3.6 mmol) in dry benzene (20 mL) was added 1,2-ethane­dithiol (1 mL) and *p*-toluenesulfonic acid (PTSA) (20 mg). The reaction mixture was refluxed and the water generated was removed with the aid of a Dean–Stark apparatus for 1 h. The progress of the reaction was monitored by TLC and at the conclusion of the reaction, the mixture was extracted with ethyl acetate (20 mL × 4). Yellow crystals were isolated when the solvent was allowed to evaporate (926 mg, 100%). The ^1^H NMR and ^13^C spectra were compared with literature reports and found to be identical. M.p. 382–383 K (literature m.p. 369–371 K; Majerski & Veljkovik, 1998).

### Refinement   

Crystal data, data collection and structure refinement details are summarized in Table 1 ▶. C-bound H atoms were positioned geometrically with C—H = 1.00 Å, and refined as rinding with *U*_iso_(H) = 1.2*U*_eq_(C).

## Supplementary Material

Crystal structure: contains datablock(s) 2, I. DOI: 10.1107/S1600536814020790/gw2148sup1.cif

Structure factors: contains datablock(s) 2. DOI: 10.1107/S1600536814020790/gw21482sup2.hkl

CCDC reference: 832292

Additional supporting information:  crystallographic information; 3D view; checkCIF report

## Figures and Tables

**Table 1:** Experimental details

Crystal data	
Chemical formula	C_13_H_14_OS_2_
*M*_r_	250.36
Crystal system, space group	Monoclinic, *P*2_1_/*n*
Temperature (K)	150
*a*, *b*, *c* (Å)	7.1332 (2), 13.9220 (3), 11.4066 (3)
β (°)	101.405 (2)
*V* (Å^3^)	1110.40 (5)
*Z*	4
Radiation type	Mo *K*α
μ (mm^−1^)	0.45
Crystal size (mm)	0.32 × 0.28 × 0.23

Data collection	
Diffractometer	Oxford Diffraction Xcalibur-S
Absorption correction	Multi-scan (*CrysAlis RED*; Oxford Diffraction, 2006 ▶)
*T*_min_, *T*_max_	0.869, 0.903
No. of measured, independent and observed [*I* > 2σ(*I*)] reflections	7821, 1963, 1828
*R*_int_	0.014
(sin θ/λ)_max_ (Å^−1^)	0.595

Refinement	
*R*[*F* ^2^ > 2σ(*F* ^2^)], *wR*(*F* ^2^), *S*	0.029, 0.082, 1.11
No. of reflections	1963
No. of parameters	145
H-atom treatment	H-atom parameters constrained
Δρ_max_, Δρ_min_ (e Å^−3^)	0.70, −0.25

Computer programs: *CrysAlis CCD* and *CrysAlis RED* (Oxford Diffraction, 2006 ▶), *SHELXS97* and *SHELXL97* (Sheldrick, 2008 ▶) and *ORTEP-3 for Windows* (Farrugia, 2012 ▶).

## supplementary crystallographic information

### Crystal data

**Table:**

C_13_H_14_OS_2_	*F*(000) = 528
*M**_r_* = 250.36	*D*_x_ = 1.498 Mg m^−^^3^
Monoclinic, *P*2_1_/*n*	Mo *K*α radiation, λ = 0.71073 Å
*a* = 7.1332 (2) Å	Cell parameters from 8769 reflections
*b* = 13.9220 (3) Å	θ = 3.4–32.7°
*c* = 11.4066 (3) Å	µ = 0.45 mm^−^^1^
β = 101.405 (2)°	*T* = 150 K
*V* = 1110.40 (5) Å^3^	Block, colorless
*Z* = 4	0.32 × 0.28 × 0.23 mm

### Data collection

**Table:**

Oxford Diffraction Xcalibur-S diffractometer	1963 independent reflections
Radiation source: Enhance (Mo) X-ray Source	1828 reflections with *I* > 2σ(*I*)
Graphite monochromator	*R*_int_ = 0.014
Detector resolution: 15.9948 pixels mm^-1^	θ_max_ = 25.0°, θ_min_ = 3.4°
ω/q–scan	*h* = −7→8
Absorption correction: multi-scan (*CrysAlis RED*; Oxford Diffraction, 2006)	*k* = −16→15
*T*_min_ = 0.869, *T*_max_ = 0.903	*l* = −13→12
7821 measured reflections	

### Refinement

**Table:**

Refinement on *F*^2^	Primary atom site location: structure-invariant direct methods
Least-squares matrix: full	Secondary atom site location: difference Fourier map
*R*[*F*^2^ > 2σ(*F*^2^)] = 0.029	Hydrogen site location: inferred from neighbouring sites
*wR*(*F*^2^) = 0.082	H-atom parameters constrained
*S* = 1.11	*w* = 1/[σ^2^(*F*_o_^2^) + (0.0452*P*)^2^ + 0.6547*P*] where *P* = (*F*_o_^2^ + 2*F*_c_^2^)/3
1963 reflections	(Δ/σ)_max_ < 0.001
145 parameters	Δρ_max_ = 0.70 e Å^−^^3^
0 restraints	Δρ_min_ = −0.25 e Å^−^^3^

### Special details

**Table:**

Experimental. Empirical absorption correction using spherical harmonics, implemented in SCALE3 ABSPACK scaling algorithm. Melting points were recorded on Labhosp or Veego melting point apparatus and are uncorrected.
Geometry. All e.s.d.'s (except the e.s.d. in the dihedral angle between two l.s. planes) are estimated using the full covariance matrix. The cell e.s.d.'s are taken into account individually in the estimation of e.s.d.'s in distances, angles and torsion angles; correlations between e.s.d.'s in cell parameters are only used when they are defined by crystal symmetry. An approximate (isotropic) treatment of cell e.s.d.'s is used for estimating e.s.d.'s involving l.s. planes.
Refinement. Refinement of *F*^2^ against ALL reflections. The weighted *R*-factor *wR* and goodness of fit *S* are based on *F*^2^, conventional *R*-factors *R* are based on *F*, with *F* set to zero for negative *F*^2^. The threshold expression of *F*^2^ > σ(*F*^2^) is used only for calculating *R*-factors(gt) *etc*. and is not relevant to the choice of reflections for refinement. *R*-factors based on *F*^2^ are statistically about twice as large as those based on *F*, and *R*- factors based on ALL data will be even larger.

### Fractional atomic coordinates and isotropic or equivalent isotropic displacement parameters (Å^2^)

**Table:**

	*x*	*y*	*z*	*U*_iso_*/*U*_eq_	
S1	0.70478 (6)	0.27978 (3)	0.98003 (4)	0.02159 (15)	
S2	0.31454 (6)	0.33911 (3)	0.86666 (4)	0.02312 (15)	
O1	0.23114 (17)	0.50405 (10)	0.65373 (11)	0.0269 (3)	
C1	0.3959 (2)	0.48249 (12)	0.69306 (15)	0.0190 (4)	
C2	0.5157 (2)	0.40985 (12)	0.64299 (15)	0.0195 (4)	
H2	0.4578	0.3809	0.5639	0.023*	
C3	0.7083 (2)	0.46561 (13)	0.65013 (15)	0.0203 (4)	
H3	0.7596	0.4710	0.5749	0.024*	
C4	0.6953 (2)	0.55972 (13)	0.72195 (15)	0.0204 (4)	
H4	0.6735	0.6198	0.6732	0.024*	
C5	0.5351 (2)	0.53335 (12)	0.79096 (15)	0.0181 (4)	
H5	0.4789	0.5901	0.8252	0.022*	
C6	0.8787 (2)	0.55754 (13)	0.81748 (16)	0.0222 (4)	
H6A	0.9956	0.5619	0.7832	0.027*	
H6B	0.8808	0.6074	0.8797	0.027*	
C7	0.8454 (2)	0.45675 (12)	0.86211 (14)	0.0172 (4)	
H7	0.9466	0.4325	0.9291	0.021*	
C8	0.6413 (2)	0.46086 (11)	0.88865 (14)	0.0157 (3)	
H8	0.6397	0.4824	0.9721	0.019*	
C9	0.5690 (2)	0.35734 (12)	0.86447 (15)	0.0164 (3)	
C10	0.6202 (2)	0.33897 (12)	0.74202 (15)	0.0176 (4)	
H10	0.6218	0.2700	0.7176	0.021*	
C11	0.8111 (2)	0.39489 (12)	0.74749 (15)	0.0183 (4)	
H11	0.9226	0.3581	0.7295	0.022*	
C12	0.5231 (3)	0.18830 (14)	0.96940 (19)	0.0299 (4)	
H12A	0.5158	0.1508	0.8949	0.036*	
H12B	0.5537	0.1438	1.0383	0.036*	
C13	0.3370 (3)	0.23778 (15)	0.9692 (2)	0.0354 (5)	
H13A	0.3324	0.2606	1.0508	0.042*	
H13B	0.2298	0.1924	0.9437	0.042*	

### Atomic displacement parameters (Å^2^)

**Table:**

	*U*^11^	*U*^22^	*U*^33^	*U*^12^	*U*^13^	*U*^23^
S1	0.0220 (2)	0.0183 (2)	0.0231 (2)	0.00132 (16)	0.00120 (17)	0.00425 (16)
S2	0.0157 (2)	0.0251 (3)	0.0290 (3)	−0.00044 (16)	0.00552 (17)	0.00363 (18)
O1	0.0195 (6)	0.0323 (7)	0.0268 (7)	0.0063 (5)	−0.0005 (5)	0.0023 (6)
C1	0.0192 (8)	0.0200 (9)	0.0177 (8)	0.0013 (7)	0.0034 (7)	0.0038 (7)
C2	0.0188 (8)	0.0235 (9)	0.0146 (8)	−0.0002 (7)	−0.0001 (6)	−0.0034 (7)
C3	0.0203 (8)	0.0271 (9)	0.0139 (8)	0.0005 (7)	0.0045 (7)	−0.0003 (7)
C4	0.0226 (9)	0.0202 (9)	0.0182 (8)	−0.0015 (7)	0.0037 (7)	0.0026 (7)
C5	0.0196 (8)	0.0160 (8)	0.0188 (8)	0.0025 (6)	0.0039 (7)	−0.0003 (6)
C6	0.0215 (9)	0.0238 (9)	0.0207 (9)	−0.0051 (7)	0.0030 (7)	0.0001 (7)
C7	0.0152 (8)	0.0207 (9)	0.0151 (8)	−0.0005 (6)	0.0016 (6)	−0.0012 (6)
C8	0.0172 (8)	0.0161 (8)	0.0139 (8)	0.0008 (6)	0.0030 (6)	−0.0014 (6)
C9	0.0143 (7)	0.0165 (8)	0.0183 (8)	0.0011 (6)	0.0029 (6)	−0.0001 (6)
C10	0.0168 (8)	0.0175 (8)	0.0183 (8)	0.0019 (6)	0.0025 (6)	−0.0045 (6)
C11	0.0158 (8)	0.0230 (9)	0.0168 (8)	0.0021 (6)	0.0046 (6)	−0.0023 (7)
C12	0.0310 (10)	0.0225 (9)	0.0371 (11)	−0.0039 (8)	0.0090 (8)	0.0072 (8)
C13	0.0351 (11)	0.0282 (10)	0.0462 (13)	−0.0019 (8)	0.0162 (10)	0.0075 (9)

### Geometric parameters (Å, º)

**Table:**

S1—C12	1.8039 (19)	C6—C7	1.527 (2)
S1—C9	1.8268 (16)	C6—H6A	0.9900
S2—C13	1.819 (2)	C6—H6B	0.9900
S2—C9	1.8375 (16)	C7—C11	1.544 (2)
O1—C1	1.210 (2)	C7—C8	1.546 (2)
C1—C2	1.507 (2)	C7—H7	1.0000
C1—C5	1.515 (2)	C8—C9	1.537 (2)
C2—C3	1.566 (2)	C8—H8	1.0000
C2—C10	1.572 (2)	C9—C10	1.533 (2)
C2—H2	1.0000	C10—C11	1.560 (2)
C3—C11	1.555 (2)	C10—H10	1.0000
C3—C4	1.558 (2)	C11—H11	1.0000
C3—H3	1.0000	C12—C13	1.495 (3)
C4—C6	1.528 (2)	C12—H12A	0.9900
C4—C5	1.555 (2)	C12—H12B	0.9900
C4—H4	1.0000	C13—H13A	0.9900
C5—C8	1.581 (2)	C13—H13B	0.9900
C5—H5	1.0000		
			
C12—S1—C9	95.59 (8)	C11—C7—H7	115.4
C13—S2—C9	98.87 (9)	C8—C7—H7	115.4
O1—C1—C2	127.43 (16)	C9—C8—C7	103.06 (12)
O1—C1—C5	127.17 (16)	C9—C8—C5	112.02 (13)
C2—C1—C5	104.77 (13)	C7—C8—C5	102.96 (12)
C1—C2—C3	101.82 (13)	C9—C8—H8	112.7
C1—C2—C10	111.87 (14)	C7—C8—H8	112.7
C3—C2—C10	89.29 (12)	C5—C8—H8	112.7
C1—C2—H2	116.6	C10—C9—C8	100.83 (13)
C3—C2—H2	116.6	C10—C9—S1	111.77 (11)
C10—C2—H2	116.6	C8—C9—S1	108.26 (11)
C11—C3—C4	103.04 (13)	C10—C9—S2	113.78 (11)
C11—C3—C2	90.48 (13)	C8—C9—S2	115.11 (11)
C4—C3—C2	107.56 (13)	S1—C9—S2	107.03 (8)
C11—C3—H3	117.3	C9—C10—C11	104.11 (13)
C4—C3—H3	117.3	C9—C10—C2	112.67 (13)
C2—C3—H3	117.3	C11—C10—C2	90.09 (12)
C6—C4—C5	104.22 (14)	C9—C10—H10	115.6
C6—C4—C3	103.11 (14)	C11—C10—H10	115.6
C5—C4—C3	101.17 (13)	C2—C10—H10	115.6
C6—C4—H4	115.5	C7—C11—C3	102.99 (13)
C5—C4—H4	115.5	C7—C11—C10	107.65 (13)
C3—C4—H4	115.5	C3—C11—C10	90.13 (12)
C1—C5—C4	100.21 (13)	C7—C11—H11	117.4
C1—C5—C8	111.96 (13)	C3—C11—H11	117.4
C4—C5—C8	102.11 (12)	C10—C11—H11	117.4
C1—C5—H5	113.7	C13—C12—S1	107.50 (14)
C4—C5—H5	113.7	C13—C12—H12A	110.2
C8—C5—H5	113.7	S1—C12—H12A	110.2
C7—C6—C4	95.07 (13)	C13—C12—H12B	110.2
C7—C6—H6A	112.7	S1—C12—H12B	110.2
C4—C6—H6A	112.7	H12A—C12—H12B	108.5
C7—C6—H6B	112.7	C12—C13—S2	108.87 (14)
C4—C6—H6B	112.7	C12—C13—H13A	109.9
H6A—C6—H6B	110.2	S2—C13—H13A	109.9
C6—C7—C11	103.84 (13)	C12—C13—H13B	109.9
C6—C7—C8	104.24 (13)	S2—C13—H13B	109.9
C11—C7—C8	100.88 (12)	H13A—C13—H13B	108.3
C6—C7—H7	115.4		
			
O1—C1—C2—C3	135.00 (18)	C5—C8—C9—S1	178.63 (10)
C5—C1—C2—C3	−36.34 (16)	C7—C8—C9—S2	−171.69 (11)
O1—C1—C2—C10	−130.97 (18)	C5—C8—C9—S2	−61.68 (16)
C5—C1—C2—C10	57.69 (17)	C12—S1—C9—C10	−95.11 (13)
C1—C2—C3—C11	112.54 (13)	C12—S1—C9—C8	154.73 (12)
C10—C2—C3—C11	0.33 (12)	C12—S1—C9—S2	30.09 (10)
C1—C2—C3—C4	8.70 (16)	C13—S2—C9—C10	115.38 (13)
C10—C2—C3—C4	−103.51 (14)	C13—S2—C9—C8	−128.95 (13)
C11—C3—C4—C6	33.71 (16)	C13—S2—C9—S1	−8.59 (11)
C2—C3—C4—C6	128.43 (14)	C8—C9—C10—C11	34.98 (15)
C11—C3—C4—C5	−73.93 (14)	S1—C9—C10—C11	−79.85 (14)
C2—C3—C4—C5	20.79 (16)	S2—C9—C10—C11	158.78 (11)
O1—C1—C5—C4	−121.01 (19)	C8—C9—C10—C2	−61.15 (15)
C2—C1—C5—C4	50.35 (15)	S1—C9—C10—C2	−175.98 (11)
O1—C1—C5—C8	131.38 (18)	S2—C9—C10—C2	62.66 (16)
C2—C1—C5—C8	−57.25 (17)	C1—C2—C10—C9	2.57 (19)
C6—C4—C5—C1	−148.84 (13)	C3—C2—C10—C9	105.03 (14)
C3—C4—C5—C1	−42.07 (15)	C1—C2—C10—C11	−102.79 (14)
C6—C4—C5—C8	−33.54 (16)	C3—C2—C10—C11	−0.33 (12)
C3—C4—C5—C8	73.22 (14)	C6—C7—C11—C3	−33.28 (15)
C5—C4—C6—C7	52.64 (15)	C8—C7—C11—C3	74.50 (14)
C3—C4—C6—C7	−52.68 (15)	C6—C7—C11—C10	−127.63 (14)
C4—C6—C7—C11	52.89 (15)	C8—C7—C11—C10	−19.86 (16)
C4—C6—C7—C8	−52.37 (15)	C4—C3—C11—C7	−0.34 (15)
C6—C7—C8—C9	149.65 (13)	C2—C3—C11—C7	−108.49 (13)
C11—C7—C8—C9	42.19 (15)	C4—C3—C11—C10	107.82 (13)
C6—C7—C8—C5	33.01 (15)	C2—C3—C11—C10	−0.34 (12)
C11—C7—C8—C5	−74.45 (14)	C9—C10—C11—C7	−9.41 (17)
C1—C5—C8—C9	−3.37 (19)	C2—C10—C11—C7	104.03 (14)
C4—C5—C8—C9	−109.75 (14)	C9—C10—C11—C3	−113.10 (13)
C1—C5—C8—C7	106.71 (15)	C2—C10—C11—C3	0.34 (12)
C4—C5—C8—C7	0.33 (15)	C9—S1—C12—C13	−47.06 (15)
C7—C8—C9—C10	−48.80 (14)	S1—C12—C13—S2	45.66 (18)
C5—C8—C9—C10	61.20 (15)	C9—S2—C13—C12	−22.26 (16)
C7—C8—C9—S1	68.63 (13)		
